# Supplementary material for: Increased General, Eating, and Body-Related Psychopathology in Inpatients in a Specialized Eating Disorders Unit after the Beginning of the COVID-19 Pandemic: A Retrospective Comparison with the Pre-Pandemic Period
Source: J Clin Med. 2023 Jan 10;12(2):573. doi: 10.3390/jcm12020573 (PMC9866300; doi:10.3390/jcm12020573)
Supplement: Supplementary file 1 [file jcm-12-00573-s001.zip › jcm-2118687-supplementary.docx]

# SUPPLEMENTARY

Table S1.. Comparison of clinical and psychopathological measures at admission and discharge after the exclusion from the post-pandemic group of the 12 individuals already present in the pre-pandemic group.

|  | Admission | | | | | Discharge | | | | |
| --- | --- | --- | --- | --- | --- | --- | --- | --- | --- | --- |
| Characteristic | pre-COVID, N=80^1^ | post-COVID, N=67^1^ | p-value^2^ | q-value^3^ | ES (95% CI)^4^ | pre-COVID, N=80^1^ | post-COVID, N=67^1^ | p-value^5^ | q-value^3^ | ES (95% CI)^4^ |
| age, years | 24.88 (8.48) | 24.98 (8.31) | >0.9 | >0.9 | 0.01 (-0.33, 0.35) |  |  |  |  |  |
| sex female | 78 (98%) | 66 (99%) | >0.9 | >0.9 |  |  |  |  |  |  |
| diagnosis |  |  | 0.14 | >0.9 |  |  |  |  |  |  |
| AN-BP | 14 (18%) | 20 (30%) |  |  |  |  |  |  |  |  |
| AN-R | 47 (59%) | 37 (55%) |  |  |  |  |  |  |  |  |
| ARFID | 3 (3.8%) | 2 (3.0%) |  |  |  |  |  |  |  |  |
| BN | 6 (7.5%) | 6 (9.0%) |  |  |  |  |  |  |  |  |
| OSFED | 10 (12%) | 2 (3.0%) |  |  |  |  |  |  |  |  |
| duration of illness,years | 6.43 (6.92) | 7.58 (8.06) | 0.4 | >0.9 | 0.15 (-0.19, 0.49) |  |  |  |  |  |
| lenght of stay, days | 34.55 (19.54) | 29.19 (19.53) | 0.13 | >0.9 | -0.27 (-0.63, 0.08) |  |  |  |  |  |
| admission from emergency room | 13 (16%) | 20 (30%) | 0.049 | 0.4 |  |  |  |  |  |  |
| BMI (underweight individuals) | 14.39 (2.21) | 14.25 (2.00) | 0.7 | >0.9 | -0.06 (-0.42, 0.29) | 15.08 (2.13) | 14.70 (1.95) | 0.3 | >0.9 | -0.18 (-0.57, 0.20) |
| caloric intake (underweight), kcal | 769.64 (499.88) | 774.90 (364.15) | >0.9 | >0.9 | 0.01 (-0.37, 0.40) | 1,457.46 (378.48) | 1,363.44 (248.84) | 0.12 | 0.7 | -0.28 (-0.67, 0.10) |
| physical hyperactivity | 24 (30%) | 36 (54%) | 0.004 | **0.036** |  | 8 (10%) | 16 (24%) | 0.023 | 0.2 |  |
| binge-purging symptoms | 18 (22%) | 21 (31%) | 0.2 | >0.9 |  | 2 (2.5%) | 2 (3.0%) | >0.9 | >0.9 |  |
| BSPS fear | 9.68 (5.05) | 9.49 (6.46) | 0.9 | >0.9 | -0.03 (-0.38, 0.31) | 7.79 (5.01) | 8.45 (6.13) | 0.5 | >0.9 | 0.12 (-0.25, 0.49) |
| BSPS avoidance | 8.95 (5.23) | 9.00 (6.71) | >0.9 | >0.9 | 0.01 (-0.34, 0.35) | 7.23 (4.83) | 8.14 (6.54) | 0.4 | >0.9 | 0.16 (-0.20, 0.53) |
| BSPS physiologic | 3.73 (2.70) | 5.86 (5.70) | 0.010 | 0.058 | 0.49 (0.14, 0.84) | 3.06 (2.91) | 4.57 (3.74) | 0.019 | 0.2 | 0.46 (0.09, 0.83) |
| BSPS total score | 22.41 (12.04) | 23.47 (16.39) | 0.7 | >0.9 | 0.07 (-0.27, 0.42) | 17.99 (11.25) | 21.10 (15.06) | 0.2 | >0.9 | 0.24 (-0.13, 0.61) |
| EDE-Q restraint | 3.14 (2.07) | 4.12 (1.83) | 0.003 | **0.023** | 0.50 (0.16, 0.83) | 1.75 (1.67) | 2.83 (1.58) | 0.002 | **0.005** | 0.66 (0.23, 1.1) |
| Unknown |  |  |  |  |  | 29 | 29 |  |  |  |
| EDE-Q eating concern | 3.01 (1.58) | 3.66 (1.38) | 0.010 | 0.058 | 0.43 (0.10, 0.77) | 2.05 (1.39) | 2.84 (1.13) | 0.004 | **0.005** | 0.61 (0.17, 1.0) |
| Unknown |  |  |  |  |  | 29 | 29 |  |  |  |
| EDE-Q shape concern | 3.98 (1.58) | 5.01 (1.39) | <0.001 | **<0.001** | 0.68 (0.34, 1.0) | 3.01 (1.93) | 4.74 (1.28) | <0.001 | **<0.001** | 1.0 (0.57, 1.5) |
| Unknown |  |  |  |  |  | 29 | 29 |  |  |  |
| EDE-Q weight concern | 3.50 (1.69) | 4.57 (1.60) | <0.001 | **0.002** | 0.64 (0.30, 1.0) | 2.46 (1.71) | 4.08 (1.47) | <0.001 | **<0.001** | 1.0 (0.55, 1.4) |
| Unknown |  |  |  |  |  | 29 | 29 |  |  |  |
| EDE-Q global score | 3.41 (1.61) | 4.34 (1.39) | <0.001 | **0.003** | 0.61 (0.27, 1.0) | 2.32 (1.52) | 3.62 (1.20) | <0.001 | **<0.001** | 0.93 (0.48, 1.4) |
| Unknown |  |  |  |  |  | 29 | 29 |  |  |  |
| STAI state anxiety | 54.32 (13.05) | 61.12 (11.17) | 0.002 | **0.014** | 0.56 (0.21, 0.90) |  |  |  |  |  |
| STAI trait anxiety | 56.90 (15.92) | 64.14 (9.96) | 0.002 | **0.014** | 0.54 (0.19, 0.88) |  |  |  |  |  |
| BDI total score | 15.97 (8.73) | 19.51 (7.54) | 0.021 | 0.085 | 0.43 (0.05, 0.80) |  |  |  |  |  |
| ^1^Mean (SD); n (%) | | | | | | | | | | |
| ^2^Welch Two Sample t-test; Fisher's exact test; Pearson's Chi-squared test | | | | | | | | | | |
| ^3^Holm correction for multiple testing | | | | | | | | | | |
| ^4^Cohen's d (95% CI). Effects can be interpreted as small for d = 0.2 , medium for d = 0.5, and large for d = 0.8. | | | | | | | | | | |
| ^5^Welch Two Sample t-test; Pearson's Chi-squared test; Fisher's exact test | | | | | | | | | | |
| Abbreviations: AN-R=Anorexia Nervosa - Restricting Type; AN-BP=Anorexia Nervosa Binge-Purging Type; ARFID=Avoidant/Restrictive Food Intake Disorder; BN=Bulimia Nervosa; OSFED=Other Specified Feeding and Eating Disorder; BMI=Body Mass Index; BSPS=Brief Social Phobia Scale; EDE-Q=Eating Disorder Examination Questionnaire; STAI=State-Trait Anxiety Inventory; BDI=Beck Depression Inventory; SD=Standard Deviation; Ci=Confidence Interval; ES=Effect-Size | | | | | | | | | | |

*Table S2. ANOVA comparing individuals with AN for diagnostic subtype and period of hospitalization.*

|  | **post** | | **pre** | | **Two-way ANOVA F** | | |
| --- | --- | --- | --- | --- | --- | --- | --- |
| **Characteristic** | **AN-BP**^1^ | **AN-R**^1^ | **AN-BP**^1^ | **AN-R**^1^ | **COVID** | **Diagnosis** | **COVID x Diagnosis** |
| physical hyperactivity | 10.00 (47.62%) | 26.00 (57.78%) | 2.00 (14.29%) | 18.00 (38.30%) | 6.36 * | 2.76 | 0.5 |
| EDE-Q restraint | 4.39 (1.64) | 3.85 (1.93) | 4.14 (1.69) | 2.65 (2.05) | 8.83 * | 6.2 * | 1.51 |
| EDE-Q eating concern | 4.05 (1.00) | 3.46 (1.33) | 3.77 (1.31) | 2.63 (1.57) | 9.17 * | 8.93 * | 0.98 |
| EDE-Q shape concern | 5.11 (1.22) | 4.88 (1.31) | 4.38 (1.39) | 3.59 (1.51) | 22.21 ** | 2.9 | 0.95 |
| EDE-Q weight concern | 4.85 (1.45) | 4.31 (1.65) | 3.99 (1.57) | 3.03 (1.62) | 18.22 ** | 5.05 * | 0.42 |
| EDE-Q global score | 4.60 (1.12) | 4.12 (1.39) | 4.07 (1.35) | 2.98 (1.55) | 16.9 ** | 6.91 * | 1.17 |
| STAI state anxiety | 61.50 (10.53) | 61.33 (11.23) | 57.08 (16.36) | 54.38 (12.55) | 7.74 * | 0.24 | 0.24 |
| STAI trait anxiety | 65.10 (7.84) | 64.21 (10.03) | 65.08 (13.07) | 55.31 (15.42) | 9.2 * | 3.35 | 3 |
| ^1^n (%); Mean (SD) | | | | | | | |
| *p<0.05, **p<0.001. Abbreviations: AN-R=Anorexia Nervosa - Restricting Type; AN-BP=Anorexia Nervosa Binge-Purging Type | | | | | | | |

Table S3. Comparison of clinical and psychopathological measures at admission and discharge between individuals hospitalized during lockdown and re-opening phases.

|  | Admission | | | | | Discharge | | | | |
| --- | --- | --- | --- | --- | --- | --- | --- | --- | --- | --- |
| Characteristic | lockdown, N=40^1^ | re-opening, N=39^1^ | p-value^2^ | q-value^3^ | ES (95% CI)^4^ | lockdown, N=40^1^ | re-opening, N=39^1^ | p-value^5^ | q-value^3^ | ES (95% CI)^4^ |
| age, years | 24.24 (6.46) | 25.24 (9.08) | 0.6 | >0.9 | 0.13 (-0.34, 0.59) |  |  |  |  |  |
| sex female | 39 (98%) | 39 (100%) | >0.9 | >0.9 |  |  |  |  |  |  |
| diagnosis |  |  | 0.5 | >0.9 |  |  |  |  |  |  |
| AN-BP | 9 (22%) | 12 (31%) |  |  |  |  |  |  |  |  |
| AN-R | 24 (60%) | 21 (54%) |  |  |  |  |  |  |  |  |
| ARFID | 0 (0%) | 2 (5.1%) |  |  |  |  |  |  |  |  |
| BN | 4 (10%) | 3 (7.7%) |  |  |  |  |  |  |  |  |
| OSFED | 3 (7.5%) | 1 (2.6%) |  |  |  |  |  |  |  |  |
| duration of illness,years | 6.16 (5.75) | 8.53 (9.17) | 0.2 | >0.9 | 0.31 (-0.16, 0.78) |  |  |  |  |  |
| lenght of stay, days | 28.94 (13.22) | 29.23 (23.07) | >0.9 | >0.9 | 0.02 (-0.48, 0.51) |  |  |  |  |  |
| admission from emergency room | 9 (22%) | 13 (33%) | 0.3 | >0.9 |  |  |  |  |  |  |
| BMI (underweight individuals) | 14.72 (1.94) | 13.91 (1.87) | 0.091 | >0.9 | -0.42 (-0.92, 0.07) | 15.16 (1.56) | 14.29 (2.00) | 0.085 | 0.7 | -0.48 (-1.0, 0.07) |
| caloric intake (underweight), kcal | 823.93 (440.53) | 750.00 (294.59) | 0.5 | >0.9 | -0.20 (-0.72, 0.33) | 1,377.14 (245.48) | 1,375.80 (254.01) | >0.9 | >0.9 | -0.01 (-0.55, 0.54) |
| physical hyperactivity | 22 (55%) | 19 (49%) | 0.6 | >0.9 |  | 11 (28%) | 6 (15%) | 0.2 | >0.9 |  |
| binge symptoms | 7 (18%) | 10 (26%) | 0.4 | >0.9 |  |  |  |  |  |  |
| bingepurging_symptoms | 9 (22%) | 16 (41%) | 0.077 | 0.8 |  | 1 (2.5%) | 1 (2.6%) | >0.9 | >0.9 |  |
| BSPS fear | 9.50 (7.09) | 9.74 (6.44) | 0.9 | >0.9 | 0.03 (-0.44, 0.51) | 7.78 (6.80) | 8.62 (5.58) | 0.6 | >0.9 | 0.13 (-0.37, 0.64) |
| BSPS avoidance | 9.03 (6.88) | 9.03 (6.27) | >0.9 | >0.9 | 0.00 (-0.47, 0.47) | 7.50 (6.70) | 8.24 (5.74) | 0.6 | >0.9 | 0.12 (-0.39, 0.62) |
| BSPS physiologic | 4.33 (4.48) | 6.79 (6.38) | 0.068 | 0.8 | 0.44 (-0.03, 0.92) | 3.19 (3.46) | 5.34 (3.51) | 0.019 | 0.2 | 0.61 (0.09, 1.1) |
| BSPS total score | 22.64 (17.67) | 24.38 (15.37) | 0.7 | >0.9 | 0.10 (-0.37, 0.58) | 18.34 (15.56) | 22.28 (13.48) | 0.3 | >0.9 | 0.27 (-0.24, 0.77) |
| EDE-Q restraint | 3.80 (1.98) | 4.23 (1.72) | 0.3 | >0.9 | 0.23 (-0.22, 0.68) | 2.69 (1.39) | 2.62 (1.73) | 0.9 | >0.9 | -0.04 (-0.64, 0.56) |
| Unknown |  |  |  |  |  | 20 | 15 |  |  |  |
| EDE-Q eating concern | 3.56 (1.41) | 3.72 (1.29) | 0.6 | >0.9 | 0.12 (-0.33, 0.57) | 2.83 (1.17) | 2.65 (1.19) | 0.6 | >0.9 | -0.15 (-0.75, 0.45) |
| Unknown |  |  |  |  |  | 20 | 15 |  |  |  |
| EDE-Q shape concern | 4.85 (1.50) | 5.11 (1.22) | 0.4 | >0.9 | 0.19 (-0.26, 0.64) | 4.42 (1.42) | 4.75 (1.40) | 0.4 | >0.9 | 0.23 (-0.37, 0.83) |
| Unknown |  |  |  |  |  | 20 | 15 |  |  |  |
| EDE-Q weight concern | 4.28 (1.72) | 4.72 (1.48) | 0.2 | >0.9 | 0.27 (-0.18, 0.72) | 3.65 (1.63) | 4.18 (1.52) | 0.3 | >0.9 | 0.33 (-0.28, 0.93) |
| Unknown |  |  |  |  |  | 20 | 15 |  |  |  |
| EDE-Q global score | 4.12 (1.49) | 4.44 (1.26) | 0.3 | >0.9 | 0.23 (-0.22, 0.68) | 3.40 (1.22) | 3.55 (1.32) | 0.7 | >0.9 | 0.11 (-0.49, 0.71) |
| Unknown |  |  |  |  |  | 20 | 15 |  |  |  |
| STAI state anxiety | 59.28 (13.40) | 61.46 (9.73) | 0.4 | >0.9 | 0.18 (-0.27, 0.64) |  |  |  |  |  |
| STAI trait anxiety | 64.18 (11.29) | 64.05 (8.66) | >0.9 | >0.9 | -0.01 (-0.47, 0.44) |  |  |  |  |  |
| BDI total score | 19.42 (8.44) | 19.76 (7.07) | 0.9 | >0.9 | 0.04 (-0.51, 0.60) |  |  |  |  |  |
| BMI |  |  |  |  |  | 16.41 (3.18) | 14.84 (2.49) | 0.033 | 0.3 | -0.54 (-1.1, -0.03) |
| caloric intake, kcal |  |  |  |  |  | 1,375.29 (229.68) | 1,371.25 (245.33) | >0.9 | >0.9 | -0.02 (-0.52, 0.49) |
| ^1^Mean (SD); n (%) | | | | | | | | | | |
| ^2^Welch Two Sample t-test; Fisher's exact test; Pearson's Chi-squared test | | | | | | | | | | |
| ^3^Holm correction for multiple testing | | | | | | | | | | |
| ^4^Cohen's d (95% CI). Effects can be interpreted as small for d = 0.2 , medium for d = 0.5, and large for d = 0.8. | | | | | | | | | | |
| ^5^Welch Two Sample t-test; Pearson's Chi-squared test; Fisher's exact test | | | | | | | | | | |
| Abbreviations: AN-R=Anorexia Nervosa - Restricting Type; AN-BP=Anorexia Nervosa Binge-Purging Type; ARFID=Avoidant/Restrictive Food Intake Disorder; BN=Bulimia Nervosa; OSFED=Other Specified Feeding and Eating Disorder; BMI=Body Mass Index; BSPS=Brief Social Phobia Scale; EDE-Q=Eating Disorder Examination Questionnaire; STAI=State-Trait Anxiety Inventory; BDI=Beck Depression Inventory; SD=Standard Deviation; Ci=Confidence Interval; ES=Effect-Size | | | | | | | | | | |

Table S4. Comparison of clinical and psychopathological measures at admission and discharge between individuals with extreme-AN hospitalized before and after the start of the COVID-19 pandemic.

|  | Admission | | | | | Discharge | | | | |
| --- | --- | --- | --- | --- | --- | --- | --- | --- | --- | --- |
| Characteristic | pre-COVID, N=34^1^ | post-COVID, N=39^1^ | p-value^2^ | q-value^3^ | ES (95% CI)^4^ | pre-COVID, N=34^1^ | post-COVID, N=39^1^ | p-value^5^ | q-value^3^ | ES (95% CI)^4^ |
| age, years | 20.91 (18.49, 26.83) | 21.29 (19.67, 27.53) | 0.4 | >0.9 | 0.20 (-0.27, 0.67) |  |  |  |  |  |
| sex female | 34 (100%) | 38 (97%) | >0.9 | >0.9 |  |  |  |  |  |  |
| admission from emergency room | 7 (21%) | 12 (31%) | 0.3 | >0.9 |  |  |  |  |  |  |
| duration of illness,years | 3.50 (1.75, 7.50) | 4.50 (1.00, 9.00) | 0.8 | >0.9 | 0.19 (-0.29, 0.67) |  |  |  |  |  |
| lenght of stay, days | 38.00 (28.00, 50.00) | 34.50 (23.75, 44.00) | 0.2 | >0.9 | -0.24 (-0.73, 0.25) |  |  |  |  |  |
| BMI | 13.20 (12.50, 13.93) | 13.04 (12.12, 13.70) | 0.9 | >0.9 | 0.06 (-0.40, 0.53) | 13.70 (13.00, 14.60) | 14.00 (12.83, 14.51) | 0.9 | >0.9 | -0.03 (-0.52, 0.45) |
| caloric intake, kcal | 550.00 (337.50, 1,000.00) | 675.00 (400.00, 900.00) | 0.6 | >0.9 | 0.00 (-0.48, 0.49) | 1,400.00 (1,300.00, 1,600.00) | 1,400.00 (1,300.00, 1,575.00) | 0.8 | >0.9 | -0.12 (-0.62, 0.37) |
| physical hyperactivity | 12 (35%) | 23 (59%) | 0.043 | 0.4 |  | 5 (15%) | 10 (26%) | 0.2 | >0.9 |  |
| binge-purging symptoms | 3 (8.8%) | 9 (23%) | 0.10 | 0.8 |  | 1 (2.9%) | 1 (2.6%) | >0.9 | >0.9 |  |
| BSPS fear | 9.50 (6.00, 13.00) | 9.50 (5.25, 15.00) | >0.9 | >0.9 | 0.03 (-0.45, 0.52) | 8.00 (5.00, 10.00) | 8.00 (5.00, 15.00) | 0.6 | >0.9 | 0.14 (-0.37, 0.66) |
| BSPS avoidance | 6.50 (5.00, 12.50) | 8.00 (3.25, 13.75) | >0.9 | >0.9 | 0.05 (-0.44, 0.54) | 6.00 (3.00, 8.00) | 7.00 (4.00, 13.50) | 0.6 | >0.9 | 0.18 (-0.33, 0.70) |
| BSPS physiologic | 3.00 (2.00, 4.25) | 3.50 (2.00, 7.00) | 0.5 | >0.9 | 0.28 (-0.21, 0.76) | 3.00 (2.00, 4.00) | 4.00 (2.00, 6.75) | 0.2 | >0.9 | 0.29 (-0.23, 0.81) |
| BSPS total score | 18.50 (12.00, 31.50) | 23.00 (9.25, 32.50) | >0.9 | >0.9 | 0.07 (-0.41, 0.56) | 16.00 (11.00, 22.00) | 21.00 (10.75, 33.00) | 0.4 | >0.9 | 0.22 (-0.30, 0.73) |
| EDE-Q restraint | 1.90 (0.35, 4.50) | 4.50 (2.15, 5.15) | 0.010 | 0.068 | 0.66 (0.18, 1.2) | 0.90 (0.25, 2.40) | 2.40 (2.00, 3.40) | 0.002 | 0.009 | 0.90 (0.29, 1.5) |
| Unknown |  |  |  |  |  | 12 | 14 |  |  |  |
| EDE-Q eating concern | 2.30 (1.15, 3.85) | 3.80 (2.55, 4.40) | 0.002 | **0.019** | 0.79 (0.30, 1.3) | 1.80 (0.60, 3.40) | 3.00 (2.60, 3.60) | 0.018 | 0.018 | 0.80 (0.20, 1.4) |
| Unknown |  |  |  |  |  | 12 | 14 |  |  |  |
| EDE-Q shape concern | 3.07 (2.32, 4.10) | 5.19 (3.78, 5.88) | <0.001 | **0.001** | 1.0 (0.53, 1.5) | 2.57 (1.16, 4.69) | 4.88 (4.13, 5.75) | 0.006 | 0.014 | 1.0 (0.35, 1.6) |
| Unknown |  |  |  |  |  | 12 | 14 |  |  |  |
| EDE-Q weight concern | 2.30 (1.60, 3.60) | 4.80 (3.40, 5.20) | 0.002 | **0.019** | 0.82 (0.32, 1.3) | 2.40 (0.80, 3.35) | 4.20 (3.00, 5.00) | 0.005 | 0.014 | 0.90 (0.29, 1.5) |
| Unknown |  |  |  |  |  | 12 | 14 |  |  |  |
| EDE-Q global score | 2.20 (1.61, 4.03) | 4.40 (3.16, 5.03) | <0.001 | **0.010** | 0.90 (0.41, 1.4) | 2.31 (0.58, 3.38) | 3.69 (2.77, 4.29) | 0.003 | 0.012 | 1.0 (0.37, 1.6) |
| Unknown |  |  |  |  |  | 12 | 14 |  |  |  |
| STAI state anxiety | 55.00 (45.25, 61.25) | 59.00 (52.00, 71.00) | 0.030 | 0.2 | 0.57 (0.07, 1.1) |  |  |  |  |  |
| STAI trait anxiety | 56.00 (40.75, 63.75) | 62.00 (56.00, 69.00) | 0.007 | 0.054 | 0.76 (0.26, 1.3) |  |  |  |  |  |
| BDI total score | 12.00 (6.50, 19.00) | 18.00 (13.62, 20.88) | 0.031 | 0.2 | 0.65 (0.10, 1.2) |  |  |  |  |  |
| ^1^Median (IQR); n (%) | | | | | | | | | | |
| ^2^Wilcoxon rank sum test; Fisher's exact test; Pearson's Chi-squared test | | | | | | | | | | |
| ^3^Holm correction for multiple testing | | | | | | | | | | |
| ^4^Cohen's d (95% CI). Effects can be interpreted as small for d = 0.2 , medium for d = 0.5, and large for d = 0.8. | | | | | | | | | | |
| ^5^Wilcoxon rank sum test; Pearson's Chi-squared test; Fisher's exact test | | | | | | | | | | |
| Abbreviations:BMI=Body Mass Index; BSPS=Brief Social Phobia Scale; EDE-Q=Eating Disorder Examination Questionnaire; STAI=State-Trait Anxiety Inventory; BDI=Beck Depression Inventory; SD=Standard Deviation; Ci=Confidence Interval; ES=Effect-Size | | | | | | | | | | |

Table S5. Comparison of body specific psychopathological measures between individuals with extreme-AN hospitalized before and after the start of the COVID-19 pandemic.

| Characteristic | pre-COVID, N=24^1^ | post-COVID, N=23^1^ | p-value^2^ | q-value^3^ | ES (95% CI)^4^ |
| --- | --- | --- | --- | --- | --- |
| BCQ specific body parts | 11.00 (8.00, 21.50) | 30.00 (14.00, 35.00) | 0.004 | **0.028** | 0.94 (0.32, 1.6) |
| BCQ idiosyncratic checking | 6.00 (5.00, 8.00) | 12.00 (8.50, 17.00) | <0.001 | **0.009** | 0.80 (0.19, 1.4) |
| BCQ total score | 32.00 (29.00, 64.00) | 74.00 (50.00, 87.00) | 0.006 | **0.032** | 0.89 (0.27, 1.5) |
| BIAQ clothing | 19.50 (13.75, 24.25) | 23.00 (20.50, 32.00) | 0.058 | 0.13 | 0.53 (-0.05, 1.1) |
| BIAQ social activities | 5.00 (2.00, 10.00) | 12.00 (7.00, 14.50) | 0.005 | **0.028** | 0.84 (0.23, 1.4) |
| BIAQ eating-related control behavior | 4.50 (0.00, 8.25) | 8.00 (5.00, 11.00) | 0.043 | 0.13 | 0.59 (0.00, 1.2) |
| BIAQ grooming/weighing | 6.50 (5.00, 9.00) | 8.00 (5.00, 10.00) | 0.4 | 0.4 | 0.23 (-0.35, 0.81) |
| BIAQ total score | 33.50 (27.50, 48.00) | 53.00 (42.50, 61.00) | 0.008 | **0.032** | 0.76 (0.16, 1.4) |
| BSQ | 79.00 (60.00, 129.00) | 153.00 (117.00, 166.00) | 0.002 | **0.012** | 1.2 (0.51, 1.8) |
| ^1^Median (IQR) | | | | | |
| ^2^Wilcoxon rank sum test | | | | | |
| ^3^Holm correction for multiple testing | | | | | |
| ^4^Cohen's d (95% CI). Effects can be interpreted as small for d = 0.2 , medium for d = 0.5, and large for d = 0.8. | | | | | |
| Abbreviations: BCQ=Body Checking Questionnaire; BIAQ=Body Image Avoidance QUestionnaire; BSQ=Body Shape Questionnaire; SD=Standard Deviation; Ci=Confidence Interval; ES=Effect-Size | | | | | |
